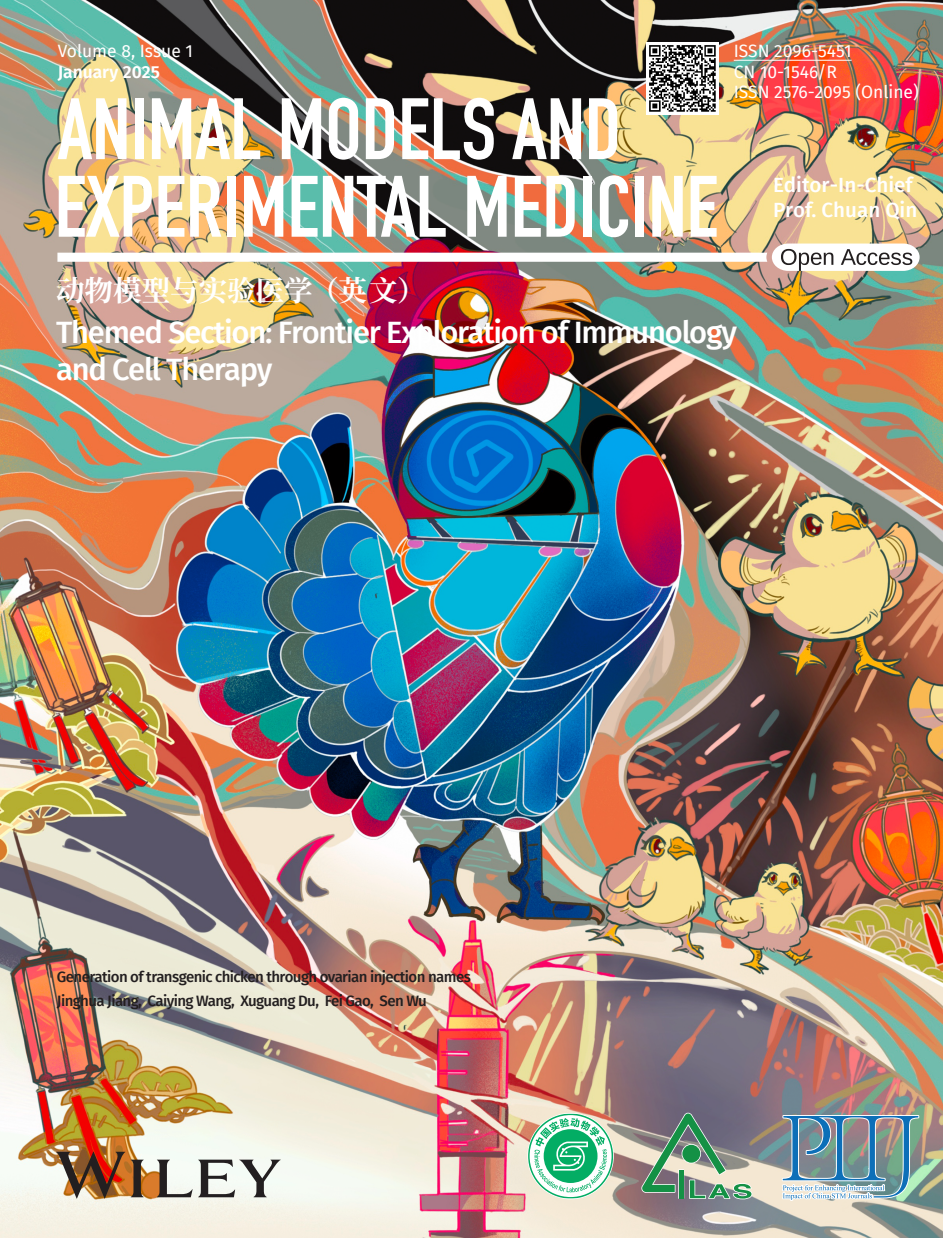# Cover Picture

**DOI:** 10.1002/ame2.12422

**Published:** 2025-02-05

## Abstract

The cover image is based on the article ‘Generation of transgenic chicken through ovarian injection’ (DOI: 10.1002/ame2.12514) reported by Jinghua Jiang, Caiying Wang, Xuguang Du, Fei Gao, Sen Wu. The article presents a technique for generating transgenic chickens in a single generation by injecting DNA into immature oocytes. This method holds immense promise for advancing the genetic manipulation capabilities of avian species that lack primordial germ cell (PGC) lines. The research not only showcases the chicken as a pivotal model organism in developmental biology but also underscores the significant value of genetic modification models in chickens.